# *Rhizocarpon geographicum* Lichen Discloses a Highly Diversified Microbiota Carrying Antibiotic Resistance and Persistent Organic Pollutant Tolerance

**DOI:** 10.3390/microorganisms10091859

**Published:** 2022-09-16

**Authors:** Alice Miral, Adam Kautsky, Susete Alves-Carvalho, Ludovic Cottret, Anne-Yvonne Guillerm-Erckelboudt, Manon Buguet, Isabelle Rouaud, Sylvain Tranchimand, Sophie Tomasi, Claudia Bartoli

**Affiliations:** 1 CNRS, ISCR (Institut des Sciences Chimiques de Rennes)—UMR 6226, University of Rennes 1, 35000 Rennes, France; 2 IGEPP, INRAE, Institut Agro, University of Rennes 1, LIPME, INRAE, 35653 Le Rheu, France; 3 CNRS, Université de Toulouse, 31320 Castanet-Tolosan, France; 4 Ecole Nationale Supérieure de Chimie de Rennes, CNRS, ISCR (Institut des Sciences Chimiques de Rennes)—UMR 6226, University of Rennes 1, 35000 Rennes, France

**Keywords:** culturomics, holobiont, antibiotic resistance, lichen microbiome

## Abstract

As rock inhabitants, lichens are exposed to extreme and fluctuating abiotic conditions associated with poor sources of nutriments. These extreme conditions confer to lichens the unique ability to develop protective mechanisms. Consequently, lichen-associated microbes disclose highly versatile lifestyles and ecological plasticity, enabling them to withstand extreme environments. Because of their ability to grow in poor and extreme habitats, bacteria associated with lichens can tolerate a wide range of pollutants, and they are known to produce antimicrobial compounds. In addition, lichen-associated bacteria have been described to harbor ecological functions crucial for the evolution of the lichen holobiont. Nevertheless, the ecological features of lichen-associated microbes are still underestimated. To explore the untapped ecological diversity of lichen-associated bacteria, we adopted a novel culturomic approach on the crustose lichen *Rhizocarpon geographicum*. We sampled *R. geographicum* in French habitats exposed to oil spills, and we combined nine culturing methods with 16S rRNA sequencing to capture the greatest bacterial diversity. A deep functional analysis of the lichen-associated bacterial collection showed the presence of a set of bacterial strains resistant to a wide range of antibiotics and displaying tolerance to Persistent Organic Pollutants (POPs). Our study is a starting point to explore the ecological features of the lichen microbiota.

## 1. Introduction

The term symbiosis was first introduced by Albert Bernhard Frank in 1876 to describe the mutualistic association between a fungal partner and a photobiont leading to a lichen symbiotic organism [1]. Lichens are widely distributed and have an incredible adaptability to highly contrasting ecosystems. They are pioneer species colonizing perturbed and extreme habitats, and they display tolerance to dryness, UV exposure, and pollutants [2]. Despite the extraordinary ecological features harbored by lichens, studies on their ecology and evolution are still in their infancy and mostly addressed to the fungal partner. Nevertheless, a recent work described the impact of algae genomic diversity on the evolutionary trajectories of a wide range of terrestrial and aquatic lichens [3]. A third partner—the lichen-associated microbiota—not strictly associated with the symbiotic interaction, has been described as a key element of the lichen evolutionary history [2,4,5,6]. Therefore, lichens have been reconsidered as holobiont units [1,7,8], a novel theory describing the host and its microbiome as a unique evolutionary entity [9]. Looking at lichens as holobionts offers new perspectives on how to explore the lichen-associated microbes and their associated ecological functions [10].

Since the beginning of the 20th Century, lichen-associated bacteria were described to harbor ecological functions crucial for the maintenance of the lichen symbiotic system [5,11,12,13,14]. Among these functions, lichen-associated bacteria display resistance activities against compounds produced by the lichenic mycobiont. For instance, several bacteria isolated from lichens were reported to resist the usnic acid produced by several lichen species [15] and exhibit a strong antimicrobial activity toward a wide range of microbes [5,16,17]. In addition, bacteria isolated from seven lichen genera showed antibacterial activities against bacterial human pathogens [18]. Indeed, lichen-associated bacteria are promising candidates in the discovery of novel antibiotics. In the context of the antibiotic resistance crisis, it is urgent to explore under both an ecological and biotechnological setting the functional properties harbored by lichen-associated microbes.

In order to dissect the ecological functions behind the microbe–lichen interaction, the culturable and the unculturable microbiota need to be deeply explored. Studies on the lichen microbiota are mostly based on molecular fingerprints [5,19,20], molecular cloning approaches [21], or 16S rRNA gene Illumina sequencing [22]. High-throughput sequencing methodologies (i.e., metabarcoding or metagenomics) are undoubtedly powerful to draw a whole picture of the lichenic microbiota. Conversely, these methodologies suffer from important limitations when applied to functional ecology [10]. Firstly, depending on the sequencing depth, microbial species can only be detected above a certain threshold. Therefore, low-abundance species with central ecological functions for the lichen can be underestimated or undetected [23]. Secondly, functional studies aiming at understanding the ecological interaction regulating the lichen holobiont are dependent on deep microbial isolation efforts. Because many host-associated microbes are uncultivable outside their habitat of origin, improving cultivability is a prerequisite for a better understanding of metacommunities interactions and their functions in complex biotic systems [10,24,25]. Culturomics is a high-throughput culturing approach combining culture methods with mass spectroscopy or 16S ribosomal RNA sequencing in order to isolate and taxonomically affiliate a wide microbial diversity. Culturomics have been extensively employed in the human gut microbiome field by expanding our knowledge on the bacterial repertoire colonizing the human gut [26]. Nevertheless, this promising approach has been unfortunately poorly utilized outside the medical field and even to a lesser extent on lichens [27].

Here, we adopted culturomics on *Rhizocarpon geographicum*, a crustose lichen pioneer of exposed rock surfaces and the first terrestrial substrate available for living organisms on Earth [28,29]. We sampled *R. geographicum* in French zones strongly affected by hostile environmental conditions (wind, sea sprays, snow). Moreover, three of the sampling locations had a history of oil spills. As a previous study revealed that the interaction of mutagenic compounds in natural environments is correlated with the development of antibiotic resistance genes in bacteria [30], we investigated the possible link between antibiotic resistance and tolerance to Persistent Organic Pollutants (POPs). We used nine culturing media mimicking the lichen habitat and two culturing methods to isolate the widest bacterial diversity. We amplified a portion of the 16S rRNA gene to affiliate the bacterial species composing *R. geographicum* microbiota. We pointed out the presence of a set of bacterial strains resistant to a wide range of antibiotics and displaying tolerance to two POPs: Methyl Tert-Butyl Ether (MTBE) and Perfluorooctanoic Acid (PFAO). Our study stresses the need to consider lichens as both sources of important ecological functions and reservoirs of biotechnologically relevant bacterial species.

## 2. Materials and Methods

### 2.1. Collection of Rhizocarpon geographicum Populations

**Figure 1:**
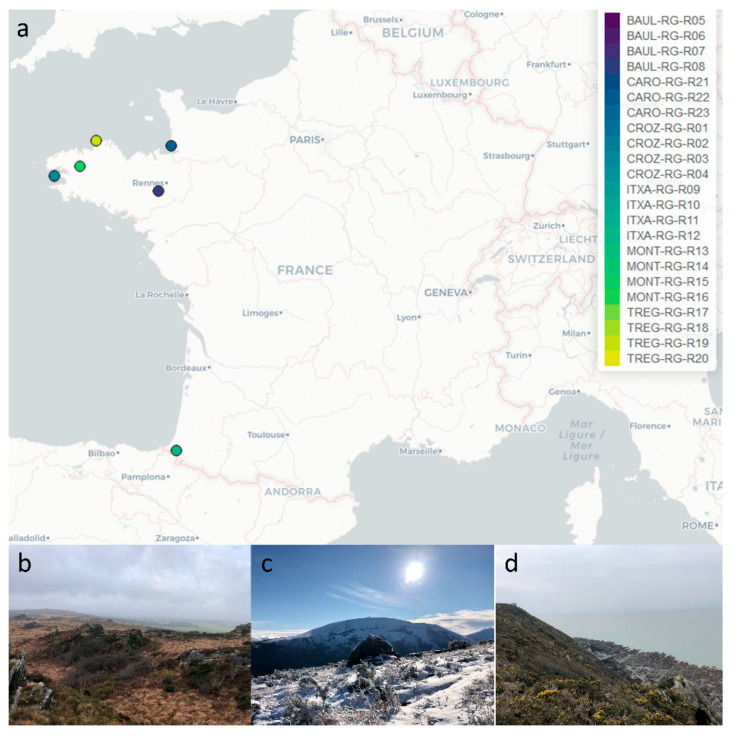
*Rhizocarpon geographicum* populations. (**a**) Locations where populations were sampled across France (north and south). Colored dots represent the *R. geographicum* populations (6 geographic sites), and the color gradient within the same localities indicates the different harvest locations. Four populations per site were sampled. (**b**) MONT populations, (**c**) ITXA populations, and (**d**) CARO populations, which are examples of the diversity of the terrestrial and maritime habitats harboring *R. geographicum*.

*R. geographicum* samples were collected in January 2021, under specific municipality authorizations, in 6 sites situated in France (Figure 1a) on: (i) the Atlantic coastal area (Ille-et-Vilaine, Finistère and Côtes d’Armor—Brittany), (ii) the English Channel coastal area in the northern limit of the Mont-Saint-Michel in Carolles (La Manche, Normandy), (iii) the island area in Baulon and Plounéour-Ménez (Ille-et-Vilaine et Finistère), and (iv) the low mountains of Itxassou (Pyrénées Atlantiques, Nouvelle Aquitaine). Sampling localities were selected based on the habitat diversity (Figure 1b–d). Three of these geographic sites (Finistère, Côtes d’Armor et La Manche) were chosen because they had a history of oil spills. For each location (called *R. geographicum* population), lichen samples were collected randomly on four rocks (Supplementary Table S1), and rock fragments were transported in sterile Petri dishes stored in individual plastic bags and processed within 6 h post collection.

### 2.2. Culturomics

Three types of matrices were prepared as organic nutrient sources and were supplemented into the media. For the first type of matrix, we developed a lichen filtrate. For this, 50 mg of *R. geographicum* was scraped and mixed with 100 mL of filtrated seawater collected from the site of sampling. The suspension was centrifuged for 10 min at 5000 rpm at 4 °C. The supernatant was recovered and sterilized by filtration through 0.22 µm pore-size Millipore^®^ membranes. The second type of organic matrix was performed on a macroalgal filtrate. For this, 155 g of *Ascophyllum nodosum* and 95 g of *Laminaria digitata* collected at la Pointe de Crozon (Britany, France) were rinsed with seawater. These algae co-habitate in the ecosystem with *R. geographicum* and are part of the lichen’s natural habitat. Macroalgae were roughly cut before being homogenized with a clean kitchen blender then bag-mixed with a Stomacher. From the concentrated solutions, 400 mL was recovered and filled with 600 mL of seawater collected at la Pointe de Crozon, previously sterilized by filtration. Once crushed, the algal pastes were passed through a stamen. Filtrates were then centrifuged for 10 min at 5000 rpm at ambient temperature. Filtrates were then filtered through 0.22 µm pore-size Millipore^®^ membranes to guarantee sterility. The third organic matrix consisted of a microalgal filtrate. Specifically, *Coccomyxa viridis*, a microalgal associated with *R. geographicum*, collected on the Atlantic coastal area in Crozon, was previously isolated on International Streptomyces Project 2 medium (ISP2, dextrose 4 g·L^−1^ (Sigma-Aldrich, St. Louis, MO, USA), yeast extract 4 g·L^−1^ (Sigma-Aldrich, St. Louis, MO, USA), malt extract 10 g·L^−1^ (Sigma-Aldrich, St. Louis, MO, USA), agar 15 g·L^−1^ (Sigma-Aldrich, St. Louis, MO, USA)). The total mass present in one Petri dish of *C. viridis* was added into 50 mL of NaCl 0.85%, then transferred into 500 mL of ISP2 liquid medium. The culture was maintained under agitation (120 rpm) for 2 weeks. After the growing time, 500 mL of filtered sea water was added into the *C. viridis* suspension and sterilized by filtration as described above.

The lichen-, macroalgal-, and microalgal-based matrices were then used to develop culturing media (Supplementary Methods). The media codes indicated in the Supplementary Methods are part of a larger list of culturing media developed at the IGEPP Laboratory (INRAE, France) with the aim to optimize culturomics on several substrates. Medium 17B (lichen–microalgal-based minimal medium) was used for bacterial enrichment, and a total of 9 media were used for direct-plating bacterial isolation (Supplementary Methods). Three media (18F, 19F, and 20F) based on lichen and/or algal suspensions were supplemented by 100 mg·L^−1^ of chloramphenicol, 300 mg·L^−1^ of streptomycin, and 100 mg·L^−1^ of penicillin G to isolate antibiotic-resistant strains. Detailed protocols for media preparation are listed in the Supplementary Methods.

### 2.3. Bacterial Isolation and Characterization

The 24 crustose lichen samples were aseptically scraped using a sterile scalpel and placed into sterile 15 mL Falcon^®^ tubes containing 5 mL of sterilized distilled water. The fresh mass of each lichen used for isolation is listed in Supplementary Table S2. To isolate the larger bacterial diversity, we adopted two methodologies. Firstly, dilutions (from 10^−1^ to 10^−3^) were directly plated on the 9 media (media supplemented with 100 mg·L^−1^ of cycloheximide: 01B, 08B, 14B, 15B; 16B, and 17B and media supplemented with 100 mg·L^−1^ of chloramphenicol, 300 mg·L^−1^ of streptomycin, and 100 mg·L^−1^ penicillin G 18F, 19F, and 20F), described in the Supplementary Material. The samples were incubated under dark conditions at 15 and 20 °C until colonies appeared (6 weeks approximately). Secondly, the 24 lichen samples were enriched in 500 mL Erlenmeyer flasks containing 50 mL of microalgae filtrate and 500 µL of lichen filtrate. The four samples for each *R. geographicum* population were pooled, and 100 µL of each lichen suspension was added to the enrichment broth. The Erlenmeyer flasks were sealed with a cotton and aluminum cap and maintained under agitation (100 rpm) at 15 °C for 4 weeks. Once a week, the cap was aseptically removed for 15 min to aerate the cultures. After 4 weeks of incubation, dilutions from the enrichment solutions were plated in triplicate on the 9 media (Supplementary Material). Plates were incubated under dark conditions at 20 °C for 6 weeks.

For both experimental procedures (direct plating and enrichment), colonies showing distinct morphologies were purified on the medium of origin and stored at −20 °C in 30% of glycerol. Pure colonies were placed into 96-well plates containing a DNA-stabilizing buffer composed of 10 mM TRIS, pH 8.0, 0.1M EDTA, pH 8.0, and 0.5% SDS. Plates were stored at −20 °C prior to DNA extraction, which was performed with the protocol described in Vingataramin and Frost (2015) [31]. Bacterial identification was performed by amplifying a region of the 16S as described in the Supplementary Material.

### 2.4. Characterization of Antibiotic-Resistant Strains

Twenty-four bacterial strains (Supplementary Table S3) able to grow on media containing antibiotics were tested for their antibiotic resistance ability against 12 antibiotics (Supplementary Table S4). For this, the 24 strains were firstly incubated in 10 mL of Tryptic Soy Broth (TSB) (Sigma, Reference T8907-1KG) from 2 to 4 days at 20 °C under agitation (120 rpm). The strains were then tested at both the exponential (*exp*) and stationary (*sta*) phase. After incubation, 10 µL of each strain at the *exp* or *sta* phase was inoculated into sterile 96-well plates containing 90 µL of TSB and the appropriate antibiotic concentration (Supplementary Table S4). TSB medium without antibiotics was used as the control. Three independent experiments (temporal blocks) were performed, and for each experiment, three replicates were performed. Plates were incubated for 72 h at 20 °C under agitation (120 rpm), then the Optical Densities (ODs) were measured at λ = 620 nm with a microplate reader Multiskan™ FC, Thermo Scientific™. The 9 values for each strain × antibiotic obtained from the 3 independent experiments were used to estimate the antibiotic effect on the bacterial growth in both the *exp* and *sta* conditions. Furthermore, the *exp* and *sta* conditions were nested into the analysis to estimate their effect on strain growth variability. For this, we first estimated an antibiotic coefficient by dividing the OD of each strain growing in the presence of the antibiotic by the OD of the strain growing in the absence of the antibiotic. Coefficient values were integrated into a generalized linear-mixed model that was run by using the *glmer* function in the lme4 R package [32,33]. Replicates and the temporal blocks were integrated in the model as random effects. *p*-values were corrected for FDR, and bar plots were built using the ggplot2 and reshape2 packages [34,35].

Nine strains showing a high antibiotic resistance range were whole-genome sequenced (Supplementary Table S3) and analyzed as described in the Supplementary Methods. Two strains that were not identified by 16S sequencing (CARO-RG-8B-R23-01 and CARO-RG-8B-R24-01) were also whole-genome sequenced to investigate their taxonomical affiliation.

### 2.5. Tolerance to Persistent Organic Pollutants

A sub-set of 394 bacterial strains belonging to unique 16S clusters and showing a suitable growth rate when re-cultured on the TSA medium after storage were tested for their ability to tolerate and grow in the presence of Persistent Organic Pollutants (POPs). For this, the 394 strains were grown for 3 days on TSA and then inoculated into TSB supplemented either with Perfluorooctanoic Acid (PFOA) at a final concentration of 20 mg·L^−1^ or Methyl Tert-Butyl Ether (MTBE) at a final concentration of 2 g·L^−1^. Controls consisted of strains inoculated on TSB only. POP screening was performed on 96-well plates containing a volume of 200 µL of TSB supplemented with the POP. Each well was inoculated with one pure bacterial colony. The inoculated 96-well plates were incubated for 7 days at room temperature, then the ODs were measured at λ = 620 nm with the microplate reader Multiskan™ FC, Thermo Scientific™. The experiment was independently repeated two times (temporal blocks), and each block was constituted by three replicates. We estimated the POP tolerance coefficient by dividing the OD of each strain growing in the presence of the given POP by the OD of the strain growing in the absence of the POP. Coefficient values were integrated into a generalized linear-mixed model that was run by using the *lmer* function implemented in the lsmeans and lme4 R packages [32,33]. The 6 replicates were considered in the model as random effects. A threshold of the POP tolerance coefficient > 1 was considered as the index of bacterial growth on the POP.

## 3. Results

### 3.1. Culturomics Revealed A Rhizocarpon geographicum Highly Diversified Microbiota

*R. geographicum* was sampled in six highly diversified maritime and terrestrial habitats located in France (Figure 1a,b). These habitats were selected to maximize niche diversity, but also to include French littorals with a history of oil spills [36]. For this, Crozon and Trégastel located in Brittany and Carolles located in Normandy were chosen to collect *R. geographicum*-associated microbes potentially harboring tolerance to hydrocarbons and pollutants. To capture the highest bacterial diversity, we applied a culturomics approach by using nine isolation media (Supplementary Methods) and two culturing methods: direct plating and enrichment with microalgae prior to plating. In order to increase the recovery of the bacterial communities, we mimicked the nutritional and environmental conditions of *R. geographicum*. Seven of the selected media consisted of algae-based and/or lichen-based media, and they were developed in our study to reproduce the *R. geographicum* habitat and isolate recalcitrant bacterial species. Three media were supplemented with antibiotics to isolate bacterial strains displaying resistance to a wide range of antibiotics. We attempted to isolate antibiotic-resistant bacteria, as Grube and collaborators identified in lichen-associated bacteria genes coding for multidrug resistance efflux pumps [11,37]. Moreover, we used antibiotics because there is molecular evidence that the presence of POPs can be implicated in the selection of microbes displaying antibiotic resistance genes in contaminated soils [30].

**Figure 2:**
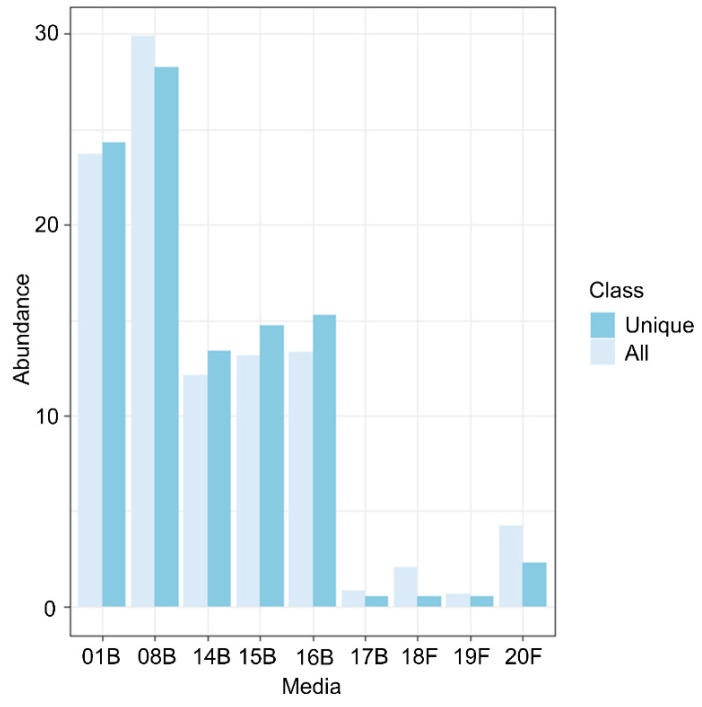
Number of bacterial strains isolated from each medium. The bar plot is based on the abundance of each bacterial strain (expressed in %) reported in the *y*-axis and calculated by dividing the number of strains recovered on each medium by the number of the total strains. The *x*-axis indicates the isolation medium. The class color scale indicates the overall isolated bacteria (light blue) and the unique bacterial clusters (dark blue) based on their 16S sequences after CD-Hist analysis.

We isolated N = 1913 bacteria from the *R. geographicum* samples. Among the 1913, we succeeded in amplifying the 16S rRNA of 1063 bacterial strains. Based on the 16S sequences, 364 bacterial clusters showing 100% sequence homology with at least one strain were identified thought the CD-HIT software [38] (Supplementary Data Set S1 and Data Set S2). The remaining 699 unique bacterial clusters (i.e., bacterial strains showing a unique 16S sequence) were taxonomically affiliated (Supplementary Data Set S2). Bacterial abundance (i.e., number of bacteria isolated per each medium divided by the total number of bacteria) showed the heterogenous success in isolating bacteria across the isolation media used (Figure 2). The highest number of strains were recovered on the 01B (nutrient agar) and the 08B (TSA) media (Figure 2). A lower number of bacterial strains were recovered from the algae- or lichen-based media (14B, 15B, 16B, 17B), and a few antibiotic resistance strains (N = 126) were isolated on the 18F, 19F, and 20F media (Figure 2). Similar results were obtained when analyzing the abundance of distinct isolated bacterial species on each medium (Supplementary Figure S1). On the other hand, media designed to reproduce the lichen environment (14B, 15B, 16B, 17B, 18F, 19F, and 20F, Supplementary Methods) allowed isolating 17 bacterial species that were not recovered on the nutrient agar (01B) and TSA (08B). Then, these bacteria were specific to the isolating medium (Table 1). Most of the species isolated from the media reproducing the lichen environment were previously reported from water reservoirs and polluted environments. For instance, *Aquaspirillum arcticum*, *Erythrobacter* sp., *Klenkia taihuensis*, *Paracoccus chinensis*, *Microterricola gilva*, and *Kocuria polaris* (Table 1) were previously isolated from marine environments [39,40,41,42], sediments [43,44], seaweed [45], or cyanobacteria [46]. It is important to note that various *Erythrobacter*, *Paracoccus*, *Kocuria*, *Pseudomonas*, or *Gordonia* species were isolated earlier from marine or maritime Brittany lichens. Furthermore, *P. helmanticensis* was isolated from an Austrian lichen [42]. *Geobacillus* sp. and *Gordonia* sp., isolated in this study on lichen-based media, were described to grow in thermal areas and polluted and contaminated environments [47,48,49,50,51,52,53]. *Geobacillus* sp. was also reported to produce antibiotics [54]. Our results suggest that media reproducing the habitat of origin help in isolating rare species that can play important roles in the ecological network of the habitat.

**Figure 3:**
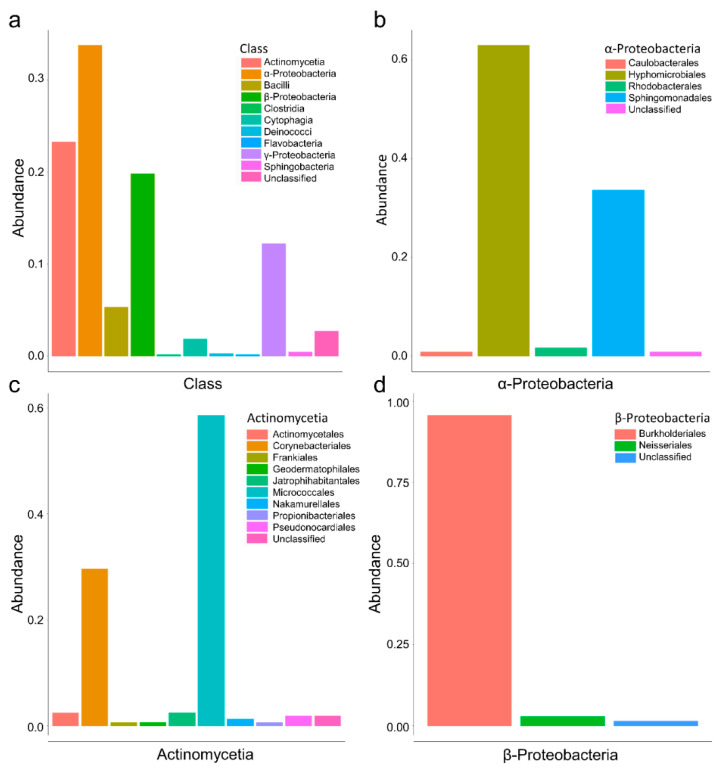
Abundance of the bacterial classes and families obtained on all isolation media. (**a**) The bar plot on the frequency (N° of each bacterial class/N° of total isolated strains) of the bacterial classes found on *Rhizocarpon geographicum*. (**b**) Bar plot on the frequency of the bacterial families included in the α-Proteobacteria class. (**c**) Bar plot on the frequency of the bacterial families included in the Actinomycetia class. (**d**) Bar plot on the frequency of the bacterial families included in the β-Proteobacteria class.

Based on the 16S taxonomy affiliation, the most abundant bacterial classes isolated by culturomics belong to α-Proteobacteria, followed by Actinomycetia, β-Proteobacteria, and γ-Proteobacteria (Figure 3a). Among the three most representative bacterial classes, the α-Proteobacteria are mostly constituted by the Hyphomicrobiales and Sphingomonadales families (Figure 3b). The Actinomycetia are mostly represented by the Micrococcales and the Frankiales orders (Figure 3c). The β-Proteobacteria are almost exclusively represented by the Burkholderiales order. These results are coherent with those obtained previously by a metagenomic study on different crustose lichens, including *R. geographicum* [20]. Therefore, the culturomics approach adopted in our study is an adequate method to explore the diversity of *R. geographicum*.

Three strains, CARO-RG-8B-R24-01, CARO-RG-8B-R23-01, and MONT-RG-14B-R14-06, did not match with the sequences in GenBank. We then extracted the total DNA of these isolates for whole-genome sequencing. For MONT-RG-14B-R14-06, the DNA did not pass the quality test and was not sequenced. Statistics on the genome quality for the two genomes is reported in Supplementary Table S5. Phylogeny on the whole genome performed by the Type Strain Genome Server (TYGS) (https://tygs.dsmz.de/, accessed on 15 July 2022) showed that the CARO-RG-8B-R23-01 strain belongs to the *Microbacterium lacticum* species; on the other hand, CARO-RG-8B-R24-01 belongs to a potential new species of the *Sphingomonas* genus (Supplementary Figure S2) distantly related to *Sphingomonas glacialis*, a psychrophilic Gram-positive bacterium isolated previously on alpine cryoconite [55].

### 3.2. Culturomics Allows Isolating Taxonomically Diversified Antibiotic-Resistant Bacterial Species

**Figure 4:**
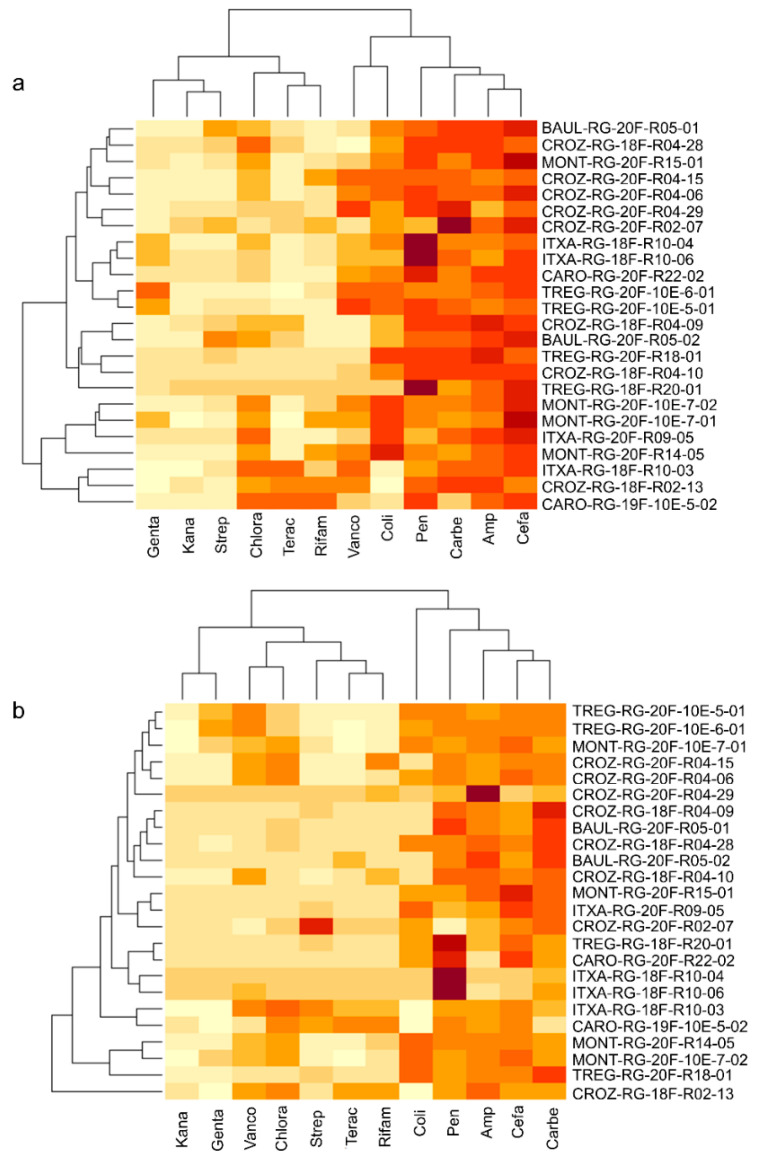
Heatmaps on the antibiotic resistance coefficient. Heatmaps were performed by lsmeans obtained by running a liner-mixed model on the antibiotic resistance coefficient and by controlling for the block and replication effect. (**a**) Heatmap on the antibiotic coefficients obtained when strains were inoculated in the stationary phase; (**b**) heatmap on the antibiotic coefficients obtained when strains were inoculated in the exponential phase. The names of the tested strains are reported in the *y*-axis; antibiotics are reported in the *x*-axis. Abbreviations: Genta = Gentamicin, Kana = Kanamycin, Vanco = Vancomycin, Chlora = Chloramphenicol, Tetrac = Tetracycline, Rifam = Rifampicin, Strep = Streptomycin, Coli = Colistin, Pen = Penicillin G, Carbe = Carbenicillin, Amp = Ampicillin, Cefa = Cefalexin. The color scale from dark red to light yellow represents high antibiotic resistance (dark red) and no resistance (light yellow).

We identified 126 bacterial strains from media containing antibiotics. Among the 126, only 87 were selected for their 16S sequence quality (Supplementary Data Set S3). Based on the phylogenetic diversity obtained on a fragment of the 16S sequence (Supplementary Figure S3), we selected 24 strains (Supplementary Table S3) for testing their antibiotic resistance degree against 12 antibiotics (Supplementary Table S4) used as therapeutics. Strains were selected to maximize their phylogenetic diversity and geographic origin. Among the 24 bacterial isolates, 10 belonged to the *Sphingomonas* genus, 8 belonged to the *Pseudomonas* genus, 3 strains were affiliated with the *Salinarimonas* genus, one strain was identified as *Paracoccus chinensis*, one was affiliated with the actinomycete *Amycolatopsis panacis*, and one strain belonged to the *Janthinobacterium* genus. The linear-mixed model performed on the antibiotic resistance coefficient showed a high effect of the strain factor for the antibiotic response (Supplementary Table S6), by confirming the heterogenicity of strains in response to the tested antibiotics (Figure 4). The *exp* and *sta* effects, determining the phase factor (whether strains were inoculated at the exponential—*exp*—or stationary—*sta*—phase), were significant on 5 of the 12 antibiotics tested (gentamicin, kanamycin, vancomycin, colistin, and cefalexin, Supplementary Table S6). On the other hand, a nested effect strain × phase factor was found for all antibiotics except penicillin G (Supplementary Table S6). This result confirms that the bacterial phase at the time of strain inoculation can influence the strain’s resistance to several antibiotic classes (Figure 4). Globally, as shown in Figure 4, resistance to antibiotics was stronger when strains were inoculated in the stationary phase. Few strains were resistant to gentamicin, kanamycin, and streptomycin (which are aminoglycoside class antibiotics inhibiting 30S bacterial ribosome subunit synthesis). For vancomycin (a glycopeptide antibiotic inhibiting bacterial cell wall biosynthesis), strains showed resistance only when inoculated in the stationary phase (Figure 4).

### 3.3. Whole-Genome Sequencing of Antibiotic-Resistant Bacteria Suggests Potential Novel Species Harboring Antimicrobial Resistance Genes

We sequenced and analyzed nine bacterial strains showing the highest degree of resistance to the tested antibiotics. The phylogenetic affiliation of the strains performed on the TYGS database [56] suggested that the nine strains are potential novel species. The TYGS database is updated daily; however, type strains closely related to the sequenced strains could be missing. Based on the phylogenetic inference on the whole genomes (Figure S4), the nine potential new species were distantly related to: (i) *Pseudomonas crudilactis* for the CROZ-RG-20F-R04-06 and CROZ-RG-20F-R04-15 strains; (ii) *Pseudomonas antartica* for the TREG-RG-20F-10-E-5-01, TREG-RG-20F-10-E-6-0, MONT-RG-20F-10-E-7-02, and MONT-RG-20F-R14-5 strains; (iii) *Sphingomonas glacialis*, *Sphingomonas panacis*, and *Sphingomonas ginsenosidivorax* for the TREG-RG-20F-R18-01, BAUL-RG-20F-R05-02 and CROZ-RG-20F-R02-07 strains, respectively (Figure S4). The closely related strains were isolated from various habitats. Indeed, *P. antarctica* and *S. glacialis* are psychrophile bacteria isolated from Antarctic water bodies [57] and alpine glacier cryoconites, respectively [55]. Both *P. antartica* and *S. glacialis* inhabit extreme environments and were described to have antimicrobial activity [55,58]. *P. crudilactis*, firstly isolated from raw milk, was shown to be resistant to several antibiotics [59]. *S. ginsenosidivorax*, isolated from ginseng [60], did not exhibit antibiotic resistance in the previous studies, but is able to bio-transform ginsenosides, possessing antimicrobial activities [61].

Analysis of the predicted KEGG metabolic pathways performed on the nine genomes and the two genomes of the strains not affiliated by 16S showed variability among the strains (Supplementary Data Set S4). The main variabilities reside in carbon degradation, nitrogen, sulfur, and hydrogen cycles, vitamins and transporters, as well as secretion systems. Cluster analysis based on the number of biological pathways and reactions showed similar results. On both the number and type of pathways and reactions, all strains were clustered in agreement with their taxonomic affiliation (Figure S5). The CARO-RG-8B-23-01 strain displayed a highly different metabolic profile, which was quite expected as belonging to an anaerobic bacterium (*Microbacterium lacticum*) (Supplementary Data Set S4, Figure S5). Interestingly, TREG-RG-20F-10E-6-01, TREG-RG-20F-10E-5-01, MONT-RG-20F-R14-05, and MONT-RG-20F-10-E-7-02, closely related to *P. antartica* (Figure S4) and carrying the strongest antibiotic resistance profile (Figure 4), showed a similar metabolic profile (Figure S5, Supplementary Data Set S4)

To better understand the genetic bases of the antibiotic resistance activity of the nine strains, we investigated the presence of Antimicrobial Resistance (AMR) proteins by using AMRFinder Plus [62], enabling accurate assessment of AMR gene content. We found that bacterial strains isolated from the Baulon lichen population carried class A β-lactamase and subclass B3 metallo-β-lactamase proteins (Supplementary Data Set S5). Strains from the Crozon lichen population displayed a wider range of AMR proteins that were annotated as efflux RND transporter permease subunit EmhB, efflux RND transporter outer membrane subunit EmhC, class C β-lactamase, and copper resistance metal-translocating P1-type ATPase CueA (Supplementary Data Set S5). Strains from the Plounéour-Ménez population were found to carry NAD(+)-rifampicin ADP-ribosyltransferase, class C β-lactamase, and fosfomycin resistance glutathione transferase proteins. Finally, strains coming from the Trégastel population and showing the highest level of antibiotic resistance were found to harbor AMR proteins annotated as fosfomycin resistance glutathione transferase, class C β-lactamase, arsinothricin resistance N-acetyltransferase ArsN1 family B, and APH(3’) family aminoglycoside O-phosphotransferase. Interestingly, the AMR profile was defined by the lichen population, suggesting that specific antimicrobial strategies are adopted at the local/habitat level.

### 3.4. Lichen Bacterial Strains Harbor Tolerance to Persistent Organic Pollutants That Is Not Correlated with the Antimicrobial Activity

In order to investigate the putative tolerance to Persistent Organic Pollutants (POPs) and the possible connection with antibiotic resistance activity, Perfluorooctanoic Acid (PFOA)—a perfluoroalkyl substance found in aqueous film-forming foams—and Methyl Tert-Butyl Ether (MTBE)—a petrol additive—were used as the sources of POPs during the bacterial growth of 394 strains (Supplementary Data Set S6). The 394 strains were selected because they were able to efficiently grow on TSA medium. Linear models demonstrated a highly significant effect of the strain factor (χ^2^ = 483.04, *p* = 0.001425) on the MTBE tolerance and on the PFOA tolerance (χ^2^ = 590.87, *p* = 4.613 × 10^−10^) by suggesting a high variability among the strains in response to both POPs tested (Supplementary Data Set S6). Regression on the LSmeans between the two POP tolerance coefficients showed an *R*^2^ = 0.11, suggesting a faint degree of correlation between both POP tolerances (Figure S6).

For the MTBE, within the 394 strains, 237 were able to tolerate the POP (tolerance coefficient ≥ 1) and 42 strains had a tolerance coefficient ≥ 1.5. The fifteen strains (3.8% of the tested bacteria) showing the highest level of tolerance (coefficient ≥ 2) were recovered from the Carolles (CARO), Crozon (CROZ), and Trégastel (TREG) populations (Supplementary Data Set S6, Table 2), localities carrying a history of oil spills. This result strongly suggests that for the MTBE, the ability of the strains to resist the tested POP is linked to the habitat of origin (Table 2). These 15 most-tolerant strains belonged to seven bacterial genera/species: (i) *Frondihabitans*, an actinobacterium known to colonize lichens; (ii) *Arthrobacter*, a ubiquitous bacterial species colonizing several substrates; (iii) *Paenibacillus*, a lichen-associated bacterium; (iv) *Amantichitinum*, a soil bacterium; (v) *Deinococcus alpinitundrae*, isolated previously from alpine environments and known to be resistant to ionizing radiation; (vi) five strains belonging to the *Pseudomonas* and *Sphingomonas* genera.

For the PFOA, 216 of the 394 tested strains showed a tolerance coefficient ≥ 1 and 39 strains a coefficient ≥ 1.5. Thirteen strains (3.2% of the tested bacteria) were highly tolerant to the PFOA, and as for the MTBE, most of them were isolated from the Carolles, Crozon, and Trégastel lichen populations (Table 2). This result reinforces a possible correlation between the habit of origin and the ability to degrade the tested POP. As for the MTBE, the most-tolerant strains belonged to the *Pseudomonas*, *Bacillus*, *Sphingomonas*, and *Paenibacillus* genera, suggesting a recurrent ability of these bacterial species to degrade a wide range of POPs. On the other hand, we also found that some of the most-tolerant strains were affiliated with the genera *Methylobacterium*, *Erythrobacter*, *Micrococcus*, and *Frigoribacterium*. In our study, POP tolerance was not correlated with antimicrobial activity, as described previously [30]. On the other hand, the POP tolerance seems to be highly distributed in habitats with an oil spill history (Table 2).

## 4. Discussion

Our novel culturomic approach to lichen-associated bacteria provides a unique picture and database on diversified bacterial communities naturally colonizing *R. geographicum*. Most of the previous culture-dependent studies on lichens only used classic media, allowing isolating a small fraction of lichen-associated microbes [42]. In addition, only two studies explored the diversity of *R. geographicum*. Firstly, Bjelland et al. [20], through a 16S rDNA metabarcoding approach, investigated the diversity of bacterial communities associated with *R. geographicum* without combining culturing methods. Secondly, a recent study from Miral et al. [63] isolated 24 bacterial strains and 68 fungal isolates from *R. geographicum* by using classical microbiological media. Miral and co-authors did not use antifungal compounds for bacterial isolation; therefore, they isolated a low bacterial diversity. For instance, most of the isolated bacteria belonged to the *Paenibacillus etheri* species. Our study is the first deep ecological investigation of bacterial communities associated with *R. geographicum*, a lichen undoubtedly difficult to sample from rock surfaces, but adapted to extreme environmental conditions. To our knowledge, only a few works have used original media enriched with lichen extract in order to isolate microbes from other lichen species [27,64]. The “Microbial Dark Matter” [65] might be recovered in laboratory conditions through a reasonable imitation of the habitat where these microbes evolve during their ecological time. Regarding this, the media developed in our study to mimic *R. geographicum* environment allowed isolating bacterial species not identified before on conventional media. Our results claim that host-specific nutrients play a substantial role in isolating rare species. Developing culturing methods on these nutrients is the missing key to build diversified microbial collections from lichens with an undiscussed importance for eco-evo studies on holobionts such as lichens. Indeed, among the 699 single clusters amplified and characterized with the 16S marker gene, 19.9% of the bacteria were identified as uncultured, including an unclassified strain and three strains without any matches when blasted on GenBank. Among the 11 whole-genome-sequenced strains, nine were putative novel species. Moreover, the long incubation time allowed recovering strains belonging to the *Frankiales* order [66], hardly isolated from lichens before. The only study on the *R. geographicum* microbial diversity, Bjelland et al. in 2011 [20], found that *R. geographicum* bacterial communities consisted mainly of Acidobacteria, Proteobacteria (α- and β-Proteobacteria), and Chloroflexi classes, with a poor discrimination at the order level due to a lack of metabarcoding resolution. In agreement with previous metabarcoding studies, by culturomics, at the bacterial class level, we also found that α-Proteobacteria and β-Proteobacteria were the predominant bacterial classes of the *R. geographicum* microbiota. In addition, we isolated a large amount of Actinomycota. Furthermore, in our study, we deeply characterized the isolated bacteria at the family and species level, and we succeeded in isolating rare species previously known to be adapted to extreme environments (Table 1).

The principal aim of our study was not only to initiate an *R. geographicum*-associated bacterial collection, but to utilize this collection to understand the ecological features of bacterial communities in relationship to their habitat of origin. As some of the strains were isolated from media containing antibiotics, we investigated their resistance degree and demonstrated that part of the lichen-associated bacteria harbor resistance to a large class of antibiotics. Reconstruction of KEGG metabolic pathways confirmed the presence of potential functions protecting bacteria against toxic compounds. For example, we found metabolic pathways related to arsenic reduction in all whole-genome-sequenced bacteria (Supplementary Data Set S4). Arsenic can be present in the environments where lichens were recovered; therefore, bacteria might metabolize or transform arsenic from the habitat as described in previous species such as *Bacillus* sp. [67]. Interestingly, the most antibiotic-resistant bacterial strains (CROZ-RG-20F-R04-06, CROZ-RG-20F-R04-15, MONT-RG-20F-10-E-7-02, MONT-RG-20F-R14-05, TREG-RG-20F-10-E-5-01, and TREG-RG-20F-10-E-6-01) carry sulfur assimilation and oxidation pathways. It has been demonstrated that inhibitors of sulfur assimilation pathways in human bacterial pathogens can act as enhancers of antibiotic therapy by suggesting a close link between sulfur metabolization and the resistance to antibiotics in bacteria [68]. Together with pathways for arsenic metabolization, sulfur assimilation could also confer to lichen-associated bacteria the ability to resist antibiotics. On the other hand, antibiotic resistance activities can emerge under extreme environmental conditions and be related to larger resistance mechanisms (i.e., resistance to pollutants or competition among microbial communities in a nutrient-poor environment).

AMR gene analyses on the 11 genomes showed resistance mechanisms for antibiotics, such as classes of β-lactamases and Resistance–Nodulation–Division (RND) efflux pumps for strains identified as potential novel species. The antibiotic resistance pathways found in these strains are common in Gram-negative bacteria [69] and well-studied in many *Pseudomonas* species [70,71]. For instance, Multidrug-Resistant (MDR) pumps play an important role during the first step of plant colonization in bacterial phytopathogens [72,73,74]. The relevance of efflux pumps in plant–bacteria interactions was also described in symbiotic bacteria. For example, mutants of *Rhizobium etli*, a mutualistic symbiont of the *Phaseolus vulgaris* bean, with a defective RmrAB efflux pump formed on average 40% less nodules than the wild-type strain [75]. Taken together, results on different habitats/hosts indicate that antibiotic resistance is a mechanism not exclusively related to protecting the bacterium against antimicrobial compounds, but also to helping the bacterium to adapt and evolve in a particular ecological context. In light of this, the lichenized fungus produces specialized metabolites with antimicrobial properties (e.g., depsidones, depsides, and dibenzofurans) [76]; these compounds are a source of abiotic stress for the bacterial communities associated with lichens. Indeed, *R. geographicum* produces a vulpinic acid derivative, rhizocarpic acid, which has shown moderate antimicrobial activity (MIC range of 32–64 µg·mL^−1^) against various multi-resistant *Staphyloccocus aureus* strains [77]. The presence of RND efflux pumps and other antimicrobial resistance pathways can then confer to the lichen bacterial microbiota the arsenal to adapt to the lichen [37]. Furthermore, the RND efflux pumps are involved in Polyaromatic Hydrocarbons’ (PAHs) degradation [78,79], a group of organic compounds which are highly toxic and contaminate terrestrial and aquatic environments. The RND-type efflux pump (EmhABC) has been described previously [78,80] as extruding hydrophobic antibiotics and PAHs, including phenanthrene, anthracene, and fluoranthene [78,81]. Taking into consideration that, in our study, *R. geographicum* was sampled in littorals with a history of oil spills, the resistome (the complex of AMR genes) might have emerged in lichen-associated bacteria in response to pollutants to help the bacteria be resilient in contaminated habitats [82,83]. In accordance with our hypothesis, several studies revealed that the genus *Pseudomonas* was dominant in PAH-contaminated sites and that hydrocarbon degradation ability and antibiotic resistance were strongly correlated in these bacteria [84]. In addition, the emergence of AMR genes was demonstrated to be 15-times higher in contaminated sites [85,86].

Our results demonstrate that, on average, 60% for MTBE and 54% for PFOA of the strains tested for POP resistance were able to grow and tolerate the POPs (tolerance coefficient ≥ 1). However, unlike what was described before, resistance to the POPs was not correlated with antibiotic resistance, when considering the most-tolerant strains (tolerance coefficient ≥ 1.5 and 2). Interestingly, strains with the highest tolerance coefficient were isolated from lichens that experienced a history of oil spills. This observation indicates that pollution and environmental constraints can select from bacterial lineages with the ability to tolerate and, probably, metabolize pollutants. Therefore, mass isolation and characterization of strains from these particular habitats can help in identifying novel microbial candidates for biotechnological purposes. In this context, culturomics needs to be developed for selecting highly pollutants-degrading strains. It is worth noticing that some POP-tolerant strains were not recovered from habitats with an oil spill history. Regarding this, as lichens are known to be extreme habitats for microbes, resistance and tolerance mechanisms might evolve without a precise pollutant selective pressure. As PAHs and the POPs tested in our study are organic substances characterized by their persistence, toxicity, mobility for long distances, and their bio-accumulative nature [87], the bacterial collection we established is relevant at a biotechnological level. In fact, currently, these compounds represent a genuine threat to wild life preservation and to human health with an urgent need to counterbalance this pollution. Bioremediation has generally been considered a sustainable approach to managing petroleum-hydrocarbon-contaminated soils. There has been an increasing focus on “green” and “sustainable” remediation, and an international standard, ISO18504:2017 Soil quality—Sustainable remediation, was recently published (2017) [82]. Based on the POP tolerance of the bacterial strains we isolated, this is a first step to developing synthetic microbial communities that are very effective in POP degradation and then a unique tool to manage the bioremediation of polluted sites.

## Figures and Tables

**Table 1:** Bacterial species isolated from lichen- and/or algal-based media. Full media description is provided in the Supplementary Methods.

Bacterial Species	Isolation Medium Code	Medium Description
*Aquaspirillum arcticum*	15B	Lichen–algal-based medium
*Geobacillus* sp.	16B	Lichen–algal-based minimal medium
*Jeongeupia chitinilytica*	15B	Lichen–algal-based medium
*Klenkia taihuensis*	14B	Macroalgae-based medium
*Kocuria polaris*	14B	Macroalgae-based medium
*Microterricola gilva*	16B	Lichen–algal-based minimal medium
*Paracoccus chinensis*	19F, 20F	Lichen–algal antibiotic based
*Pseudomonas gessardii*	20F	Lichen–algal antibiotic based
*Pseudomonas helmanticensis*	16B, 20F	Lichen–algal-based medium and lichen–algal antibiotic based
*Roseomonas mucosa*	15B	Lichen–algal-based medium
*Sinomonas* sp.	15B	Lichen–algal-based medium
*Skermanella* sp.	14B	Macroalgae-based medium
*Tardiphaga* sp.	14B	Macroalgae-based medium
uncultured *Erythrobacter* sp.	16B	Lichen–algal-based minimal medium
uncultured *Gordonia* sp.	16B	Lichen–algal-based minimal medium
uncultured *Nakamurella* sp.	8B, 16B	TSA and lichen–algal-based minimal medium
*Zafaria cholistanensis*	14B	Macroalgae-based medium

**Table 2:** Percentage of *R. geographicum*-associated bacteria highly tolerant to both MTBE and PFOA (when considering a tolerance coefficient ≥ 2). na indicates that any strain from the specific locality was identified with a tolerance coefficient ≥ 2. Localities indicated in bold correspond to habitats that have been subjected to oil spills in their history.

Lichen Population	Locality	% of Tolerant Strains for MTBE	% of Tolerant Strains for PFOA
BAUL	Baulon	na	0.76
**CARO**	Carolles	1.01	1.2
**CROZ**	Crozon	1.07	0.76
ITXA	Itxassou	0.25	0.25
MONT	Plounéour-Ménez	na	na
**TREG**	Trégastel	0.7	0.5

## Data Availability

Raw datasets and 16s sequences of the strains are available in the Supplementary Data Set. Whole-genome sequences are available under the GenBank numbers reported in Supplementary Data Set S3.

## Supplementary Materials

The following Supporting Information can be downloaded at: https://www.mdpi.com/article/10.3390/microorganisms10091859/s1, Supplementary Information including Supplementary Methods, tables, and figures. Supplementary Data Set S1. Bacterial clusters sharing 100% sequence homology with two or more strains after CD-HIT analysis. Supplementary Data Set S2. Taxonomy of the 696 bacterial unique clusters identified after CD-HIT trimming. Supplementary Data Set S3. 16S sequences of strains isolated on media containing antibiotics. Supplementary Data Set S4. Heatmap of the KEGG metabolic pathways found on the strains that were whole-genome-sequenced. Antibiotic-resistant strains are marked with “*”. Color gradient from white to red reflects the completeness of the metabolic pathway. Supplementary Data Set S5. Protein classes found by using the AMRFinder Plus pipeline to be associated with antibiotic resistance or resistance to heavy metals. The analysis was performed on the nine genomes of strains described to carry a broad spectrum of resistance to antibiotics. Protein sequences are reported. Supplementary Data Set S6. LSmeans estimated on the POP tolerance of each strain. Table S1. Names and GPS coordinates (expressed in degrees) of the 24 sites on which *Rhizocarpon geographicum*. Table S2. Mass of *R. geographicum* lichen used for bacterial isolation. Table S3. Bacteria isolated on media containing antibiotics and used for the antibiotic susceptibility test. Taxonomic affiliation was assigned by blastn. Table S4. Antibiotics and relative concentrations (µg/mL) used for the antibiotic susceptibility test. Table S5. Statistics of clean genomic data obtained after fastq trimming. Table S6. Analysis of Deviance Table (Type II Wald chisquare tests) on the antibiotic resistance coefficient of the 24 strains selected for antibiotic resistance assays. Phase indicates the exponential or stationary phase at which strains have been tested. The phase and the strain *factor* were analyzed as fixed variables. *p* value adjustment was estimated with Tukey method for comparing a family of 48 estimates. Figure S1. Number of species isolated for each medium. Bar plot is based on the abundance of each bacterial species (expressed in %) calculated by dividing the number of species recovered on each medium by the number of the total species recovered. Bleu scale color represents the different media used for bacterial isolation (see Supplementary Methods). Bar plot clearly shows that as observed for the abundance of the bacterial isolated for each medium, the 01B and 08B media recovered the maximum of the bacterial diversity. Figure S2. Phylogenetic tree based on the whole genome of the CARO-RG-8B-R24-01 and CARO-RG-8B-R23-01 strains obtained by the Type Strain Genome Service (TYGS). Figure S3. Phylogenetic tree based on the 16S sequences of strains isolated from media containing antibiotics. Figure S4. Phylogeny representing CROZ-RG-20F-R04-06, CROZ-RG-20F-R04-15, MONT-RG-20F-10-E-7-02, MONT-RG-20F-R14-05, TREG-RG-20F-10-E-5-01 and TREG-RG-20F-10-E-6-01 bacterial strains (a) and BAUL-RG-20F-R05-02, CARO-RG-8B-R24-01, CROZ-RG-20F-R02-07 and TREG-RG-20F-R18-01 (b) bacterial strains. Phylogenetic tree was inferred with FastMe 2.1.6.1 on the TYGS platform [56] from GBDP distances calculated from whole-genomes. The tree was rooted ad midpoint. Strains isolated from *R. geographicum* and the related tree branches are indicated in blue. Figure S5. Network analysis based on genome functional annotation. Number of pathways (top left barplot) and number of reactions (top right barplot) based on the annotations of the 11 genomes. Clustering analyses based on the presence/absence of biological pathways (bottom left) and biological reactions (bottom right). The functional clustering is coherent with the taxonomical annotation of the strains. Plots were performed thanks to the MeCompR shiny application. Figure S6. Correlation between Lsmeans values obtained from the POP tolerance experiment. *x*-axis represents Lsmeans values obtained from strains grew in presence of PFOA and *y*-axis represents Lsmeans values obtained from strains grew in presence of MTBE. The *R^2^* representing the correlation between the two variables is also reported. References [34,38,56,88,89,90,91] are cited in the supplementary materials.
