# Supplementary material for: Rhizocarpon geographicum Lichen Discloses a Highly Diversified Microbiota Carrying Antibiotic Resistance and Persistent Organic Pollutant Tolerance
Source: Microorganisms. 2022 Sep 16;10(9):1859. doi: 10.3390/microorganisms10091859 (PMC9503503; doi:10.3390/microorganisms10091859)
Supplement: Supplementary file 1 [file microorganisms-10-01859-s001.zip › Miral et al Supp Info_vf_submission.pdf]

Supplementary Information for:

Rhizocarpon geographicum lichen discloses a highly diversified microbiota carrying antibiotic resistance and Persistent Organic Pollutants tolerance

Alice Miral[1], Adam Kautsky[2], Susete Alves-Carvalho[2], Ludovic Cottret[3], Anne-Yvonne Guillerm-Erckelboudt[2], Manon Buguet[1], Isabelle Rouaud[1], Sylvain Tranchimand[4], Sophie Tomasi[1] and Claudia Bartoli[2*]

[1] Univ Rennes, CNRS, ISCR (Institut des Sciences Chimiques de Rennes) – UMR 6226, F-35000 Rennes, France.
[2] IGEPP, INRAE, Institut Agro, Univ Rennes, 35653, Le Rheu, France LIPME, INRAE.
[3] CNRS, Université de Toulouse, Castanet-Tolosan, France.
[4] Univ Rennes, Ecole Nationale Supérieure de Chimie de Rennes, CNRS, ISCR (Institut des Sciences Chimiques de Rennes) - UMR 6226, F-35000 Rennes, France.

[*]To whom correspondence may be addressed to:

Claudia Bartoli
Phone: +33 2 23 48 51 96
Email: claudia.bartoli-kautsky@inrae.fr

This file includes:

Supplementary Methods
Supplementary Tables
Supplementary Figures

SUPPLEMENTARY METHODS

Media used for bacteria isolation from *R. geographicum*

Media codes are indicated into parentheses after the medium name. This code has been added to the bacterial strains and it represents a code belonging to a larger list of media developed at the IGEPP Laboratory (INRAE, Bretagne, France).

Product references are reported to allow the repeatability of the preparation.

Nutrient Agar (01B)

Per Liter of medium:

Osmotic water	1 L
Beef extract (VWR, Reference: 1.03979.0500 500 g)	3.0 g
Peptone (GICO, Reference: 211677 500 g)	5.0 g
Glucose (Sigma, Reference: 16301 1 kg)	1.0 g
Yeast extract (BD, Reference 212750 500 g)	0.5 g
Bacto Agar (BD, Reference 214010 500 g)	15 g

After autoclaving, add 100 mg.L-1 of cycloheximide (Sigma, Reference: 239764).

Tryptic Soy Agar Minimal (08B)

Per Liter of medium:

Osmotic water	1 L
Tryptic Soy Broth (Sigma, Reference T8907-1 kg)	3 g
Bacto Agar (BD, Reference 214010 500 g)	15 g

After autoclaving, add 100 mg.L-1 of cycloheximide (Sigma, Reference: 239764).

Macroalgae-based medium (14B)

Per Liter of medium:

Osmotic water	900 mL
K_2HPO_4, (Sigma, Reference: P3786 500 g)	3.5 g
KH_2PO_4, (VWR, Reference: 1 kg)	1.0 g
$MgSO_4$ *$7H_2O$, (Sigma, Reference: 230391 1 kg)	1.5 g
Glucose (Sigma, Reference: 16301 1 kg)	0.5 g
Bacto Agar (BD, Reference 214010 500 g)	15 g

check pH (~6)

After autoclaving, add 50 mL of each macroalgae filtrate (50 ml of the *Ascophyllum nodosum* filtrate and 50 mL osf the *Laminaria digitata* filtrate) described in the Material and Methods section of the main text. Also add 100 mg.L-1 of cycloheximide (Sigma, Reference: 239764).

Lichen algal-based medium (15B)

<u>Per Liter of medium:</u>

Osmotic water	900 mL
K_2HPO_4, (Sigma, Reference: P3786 500 g)	3.5 g
KH_2PO_4, (VWR, Reference: 1 kg)	1.0 g
$MgSO_4$ *$7H_2O$, (VWR, Reference: 1 kg)	1.5 g
Glucose Sigma, Reference: 16301 1 g)	0.5 g
Bacto Agar (BD, Reference 214010 500 g)	15 g

check pH (~6)

After autoclaving, add 50 mL of each macroalgae filtrate (50 ml of the *Ascophyllum nodosum* filtrate and 50 mL of the *Laminaria digitata* filtrate) described in the Material and Methods section of the main text. In addition, add 5 ml of lichen filtrate prepared as described in the main text. Also add 100 mg.L-1 of cycloheximide (Sigma, Reference: 239764).

Lichen algal-based minimal medium (16B)

<u>Per Liter of medium:</u>

Osmotic water	800 mL
Sea water	95 mL
Bacto Agar (BD, Reference 214010 500 g)	15 g

check pH (~6)

After autoclaving, add 50 mL of each macroalgae filtrate (50 ml of the *Ascophyllum nodosum* filtrate and 50 mL of the *Laminaria digitata* filtrate) described in the Material and Methods section of the main text. In addition, add 5 mL of lichen filtrate prepared as described in the main text. Also add 100 mg.L-1 of ycloheximide (Sigma, Reference: 239764).

Lichen microalgal-based minimal medium (17B)

<u>Per Liter of medium:</u>

Osmotic water	850 mL
Sea water	95 mL
Bacto Agar (BD, Reference 214010 500 g)	15 g

check pH (~6)

After autoclaving, add 50 mL of the *Coccomyxa viridis* filtrate obtained as described in the material and methods section of the main text. In addition, add 5ml of lichen filtrate prepared as described in the main text. Also add 100 mg.L-1 of cycloheximide (Sigma, Reference: 239764).

Algae antibiotic-based medium (18F)

<u>Per Liter of medium:</u>

Osmotic water	800 mL
Papaic Digest of Soybean Meal (Gibco, Reference 2436 500 g)	2.0 g
Dextrose (Glucose $C_6H_{12}O_6$) (Sigma, Reference 16301 1 kg)	1.0 g
Sea water	100 ml
Bacto Agar (BD, Reference 214010 500 g)	15 g

check pH (~6)

After autoclaving, add 50 mL of each macroalgae filtrate (50 mL of the *Ascophyllum nodosum* filtrate and 50 mL of the *Laminaria digitata* filtrate) described in the Material and Methods section of the main text. Also**,** add 100 mg.L-1 of chloramphenicol (Sigma, Reference: C0378-100g), 300 mg.L-1 of streptomycin (Sigma, Reference: S6501) and 100 mg.L-1 penicillin (Sigma, Reference: 1504489).

Lichen algal antibiotic-based minimal medium (19F)

Per Liter of medium:

Osmotic water	800 mL
Sea water	95 ml
Bacto Agar (BD, Reference 214010 500 g)	15 g

check pH (~6)

After autoclaving, add 50 mL of each macroalgae filtrate (50 mL of the *Ascophyllum nodosum* filtrate and 50 mL of the *Laminaria digitata* filtrate) described in the Material and Methods section of the main text. In addition, add 5 mL of lichen filtrate prepared as described in the main text. Also**,** add 100 mg.L-1 of chloramphenicol (Sigma, Reference: C0378-100g), 300 mg.L-1 of streptomycin (Sigma, Reference: S6501) and 100 mg.L-1 penicillin (Sigma, Reference: 1504489).

Lichen algal antibiotic-based medium (20F)

Osmotic water	800 mL
Mycological peptone (Sigma 77199-500 g)	5 g
Magnesium sulphate ($MgSO4$) (Sigma, Reference: 77199-500 g)	2.5 g

check pH (~6)

After autoclaving, add 50 mL of each macroalgae filtrate (50 mL of the *Ascophyllum nodosum* filtrate and 50 mL of the *Laminaria digitata* filtrate) described in the Material and Methods section of the main text. In addition, add 5 mL of lichen filtrate prepared as described in the main text. Also**,** add 100 mg.L-1 of chloramphenicol (Sigma, Reference: C0378-100g), 300 mg.L-1 of streptomycin (Sigma, Reference: S6501) and 100 mg.L-1 penicillin (Sigma, Reference: 1504489).

Bacterial identification

To characterize the bacterial strain collection, we amplified a portion of the 16S rRNA gene. Universal primers 515F (5′-GTGCCAGCMGCCGCGGTAA-3′) and 806R (5′-GGACTACVSGGGTATCTAAT-3′) [1] were used in 24 µL of PCR mix performed with 5X GoTaq® DNA Polymerase (Promega) kit. 2 µL of the extracted DNAs were added in the mix. PCR conditions are as follows: 95°C for 5 min and 35 cycles at 95°C for 5 s, 55°C for 30 s, 72°C for 60 s with a final extension at 72°C for 5 min. PCR amplicons were verified by loading 5 µL of each product on a 1 % w/v agarose gel. Products showing the suitable band size were then purified by adding 0.5 µL of Antarctic phosphatase, 0.5 µL of Exonuclease I and 1 µL of Phosphatase Buffer to 10 µL of PCR product. Sample mixtures were then incubated for 30 min at 37°C and 10 min at 90°C to deactivate the enzyme. Purified PCR products were sequenced at the Macrogen Europe BC (Amsterdam, Netherlands). Chromatograms were individually analyzed with Chromas Lite v 2.6.6 (Technelysium Ltd.) was used for manual corrections of the obtained raw sequences. and used to build a multi-FASTA file. To remove the clonal sequences and group unique sequence clusters, we run the Cd-hit software [2] with the following parameters -c 1 -aS 1 -d 50000 -T 10. Unique bacterial clusters were then integrated into Blastn with parameters: -evalue 0.000001 -num_threads 10 -max_target_seqs 20 -outfmt. A homemade perl script was then used to reconstruct a taxonomy table represent the phylogenetic affiliation of the strains. Bar plots representing the diversity of the bacterial strains were performed with ggplot2 [3].

Whole-genome Sequencing and bioinformatics

Nine bacterial strains showing the highest antibiotic resistance profile (Supplementary Table S3) and two strains whose 16S sequence fragment did not match in GenBank (CARO-RG-8B-R23-01 and CARO-RG-8B-R24-01) were whole genome sequenced. Prior DNA extraction, strains were grown from 2 to 4 days (depending on the growth rate of the strain) in TSB medium. At the stationary phase, bacterial cultures were centrifuged for 10 min at 5000 rpm and the supernatant was discarded. Bacterial pellet was then washed in sterilized MQ water and further centrifuged for 10 min. The collected pellet was then employed for DNA extraction by using the DNeasy Blood & Tissue Kit of Qiagen. A RNase step (with the Invitrogen™ RNase A 20 mg/mL) was added to the kit protocol to obtain RNA-free DNAs. For this 5 µL of RNaseA were added into the samples that were incubated at 65°C for 20 min. The concentration and purity of DNAs were estimated with spectometry. DNA degradation level was checked by running 3 µL of the extracted DNAs on a 1 % w/v agarose gel. Genomic DNA libraries were performed with the Short-Insert Library protocol and sequenced with the DNBseq™ platform (PE150 for sequencing length) at the BGI sequencing facility (Shenzhen, China).

Raw reads in the Fastq file format were filtered by removing adaptor sequenced, contaminations and low-quality reads by BGI. Clean reads were then annotated and assembled by using the Bacannot framework https://bacannot.readthedocs.io/en/latest/#index--page-root. KEGG KO annotation was performed with KofamScan version 1.3.0 [4]. Taxonomy affiliation on the whole genome sequences was performed with TYGS (Type Strain Genome Server) https://tygs.dsmz.de/ [5].

Metabolic functional analysis on the nine bacterial genomes was performed. First, for each bacterial genome, a list of biochemical reactions was inferred from its proteic sequences with Carveme software [6]. Then, each reaction was classified into metabolic pathways by querying the BIGG database [7]. To compare the presence of metabolic pathways and reactions,

presence-absence matrices were computed thanks to ad-hoc programs developed with the Met4j library [1].

.

[1] https://forgemia.inra.fr/metexplore/met4j

SUPPLEMENTARY TABLES

Supplementary Table S1. Names and GPS coordinates (expressed in degrees) of the 24 sites on which *Rhizocarpon geographicum*.

Population name	Locality	Altitude (m)	Lattitude	Longitude
CROZ-RG-R01	Crozon	30	48.2333333	-4.565277777777
CROZ-RG-R02	Crozon	50	48.2330556	-4.56638888888888
CROZ-RG-R03	Crozon	40	48.2319444	-4.57027777777777
CROZ-RG-R04	Crozon	10	48.2336111	-4.56555555555555
BAUL-RG-R05	Baulon	60	47.9713889	-1.8975
BAUL-RG-R06	Baulon	60	47.9713889	-1.8975
BAUL-RG-R07	Baulon	80	47.9722222	-1.89749972222222
BAUL-RG-R08	Baulon	80	47.9725	-1.89749972222222
ITXA-RG-R09	Itxassou	610	43.3052778	-1.42888888888888
ITXA-RG-R10	Itxassou	610	43.3025	-1.42805555555555
ITXA-RG-R11	Itxassou	620	43.3022222	-1.42805555555555
ITXA-RG-R12	Itxassou	660	43.3016667	-1.4325
MONT-RG-R13	Plounéour-Ménez	390	48.4055556	-3.90999999999999
MONT-RG-R14	Plounéour-Ménez	370	48.8369444	-3.50388888888888
MONT-RG-R15	Plounéour-Ménez	380	48.4058333	-3.90944444444444
MONT-RG-R16	Plounéour-Ménez	380	48.4055556	-3.90916666666666
TREG-RG-R17	Trégastel	10	48.8366667	-3.50388888888888
TREG-RG-R18	Trégastel	20	48.8369444	-3.50388888888888
TREG-RG-R19	Trégastel	20	48.8372222	-3.5
TREG-RG-R20	Trégastel	10	48.8372222	-3.50194444444444
CARO-RG-R21	Carolles	70	48.7441667	-1.56944444444444
CARO-RG-R22	Carolles	60	48.7444444	-1.56972222222222
CARO-RG-R23	Carolles	60	48.7463889	-1.57055555555555
CARO-RG-R24	Carolles	60	48.7461111	-1.57055527777777

Supplementary Table S2. Mass of *R. geographicum* lichen used for bacterial isolation.

Population name	Mass (mg)
CROZ-RG-R01	80.2
CROZ-RG-R02	250
CROZ-RG-R03	72.9
CROZ-RG-R04	53.5
BAUL-RG-R05	33
BAUL-RG-R06	80
BAUL-RG-R07	33.9
BAUL-RG-R08	40.1
ITXA-RG-R09	28.9
ITXA-RG-R10	37.4
ITXA-RG-R11	43.9
ITXA-RG-R12	27.4
MONT-RG-R13	30.2
MONT-RG-R14	32.8
MONT-RG-R15	37.6
MONT-RG-R16	16.5
TREG-RG-R17	164.7
TREG-RG-R18	250.7
TREG-RG-R19	525.4
TREG-RG-R20	449.6
CARO-RG-R21	42.2
CARO-RG-R22	36.2
CARO-RG-R23	48.4
CARO-RG-R24	45.4

Supplementary Table S3. Bacteria isolated on media containing antibiotics and used for the antibiotic susceptibility test. Taxonomic affiliation was assigned by blastn.

Codes	Class	Order	Family	Genus	Species	Whole genome sequences strains and related NCBI code
BAUL-RG-20F-R05-01	Actinomycetia	Pseudonocardiales	Pseudonocardiaceae	Amycolatopsis	*Amycolatopsis panacis*	no
BAUL-RG-20F-R05-02	Alphaproteobacteria	Sphingomonadales	Sphingomonadaceae	Sphingomonas	uncultured *Sphingomonas* sp.	JAKNQH000000000
CARO-RG-19F-E-10-5-02	Betaproteobacteria	Burkholderiales	Oxalobacteraceae	Janthinobacterium	uncultured *Janthinobacterium* sp.	no
CARO-RG-20F-R22-02	Alphaproteobacteria	Sphingomonadales	Sphingomonadaceae	Sphingomonas	uncultured *Sphingomonas* sp.	no
CROZ-RG-18F-R02-13	Gammaproteobacteria	Pseudomonadales	Pseudomonadaceae	Pseudomonas	*Pseudomonas* sp.	no
CROZ-RG-18F-R04-09	Alphaproteobacteria	Sphingomonadales	Sphingomonadaceae	Sphingomonas	*Sphingomonas* sp.	no
CROZ-RG-18F-R04-10	Alphaproteobacteria	Sphingomonadales	Sphingomonadaceae	Sphingomonas	*Sphingomonas* sp.	no
CROZ-RG-18F-R04-28	Alphaproteobacteria	Sphingomonadales	Sphingomonadaceae	Sphingomonas	*Sphingomonas* sp.	no
CROZ-RG-20F-R02-07	Alphaproteobacteria	Sphingomonadales	Sphingomonadaceae	Sphingomonas	*Sphingomonas* sp.	JAKNQJ000000000
CROZ-RG-20F-R04-06	Gammaproteobacteria	Pseudomonadales	Pseudomonadaceae	Pseudomonas	*Pseudomonas helmanticensis*	JAKNQK000000000
CROZ-RG-20F-R04-15	Gammaproteobacteria	Pseudomonadales	Pseudomonadaceae	Pseudomonas	*Pseudomonas frederiksbergensis*	JAKNQL000000000
CROZ-RG-20F-R04-29	Alphaproteobacteria	Rhodobacterales	Rhodobacteraceae	Paracoccus	*Paracoccus chinensis*	no
ITXA-RG-18F-R10-03	Alphaproteobacteria	Hyphomicrobiales	Salinarimonadaceae	Salinarimonas	*Salinarimonas* sp. Ri3Pw_6150	no
ITXA-RG-18F-R10-04	Alphaproteobacteria	Hyphomicrobiales	Salinarimonadaceae	Salinarimonas	*Salinarimonas* sp. Ri3Pw_6150	no
ITXA-RG-18F-R10-06	Alphaproteobacteria	Hyphomicrobiales	Salinarimonadaceae	Salinarimonas	*Salinarimonas* sp. Ri3Pw_6150	no
ITXA-RG-20F-R09-05	Alphaproteobacteria	Sphingomonadales	Sphingomonadaceae	Sphingomonas	*Sphingomonas* sp.	no
MONT-RG-20F-E-10-7-01	Gammaproteobacteria	Pseudomonadales	Pseudomonadaceae	Pseudomonas	uncultured *Pseudomonas* sp.	no
MONT-RG-20F-E-10-7-02	Gammaproteobacteria	Pseudomonadales	Pseudomonadaceae	Pseudomonas	uncultured *Pseudomonas* sp.	JAKNQN000000000
MONT-RG-20F-R14-05	Gammaproteobacteria	Pseudomonadales	Pseudomonadaceae	Pseudomonas	*Pseudomonas gessardii*	JAKNQM000000000

MONT-RG-20F-R15-01	Alphaproteobacteria	Sphingomonadales	Sphingomonadaceae	Sphingomonas	uncultured *Sphingomonas* sp.	no
TREG-RG-18F-R20-01	Alphaproteobacteria	Sphingomonadales	Sphingomonadaceae	Sphingomonas	uncultured *Sphingomonas* sp.	no
TREG-RG-20F-E-10-5-01	Gammaproteobacteria	Pseudomonadales	Pseudomonadaceae	Pseudomonas	uncultured *Pseudomonas* sp.	JAKNQO000000000
TREG-RG-20F-E-10-6-01	Gammaproteobacteria	Pseudomonadales	Pseudomonadaceae	Pseudomonas	*Pseudomonas* sp.	JAKNQP000000000
TREG-RG-20F-R18-01	Alphaproteobacteria	Sphingomonadales	Sphingomonadaceae	Sphingomonas	*Sphingomonas melonis*	JAKNQQ000000000

Supplementary Table S4. Antibiotics and relative concentrations (µg/mL) used for the antibiotic susceptibility test.

**Antibiotics**	**Concentration (µg/mL)**
Gentamicin	5
Ampicillin	100
Streptomycin	150
Kanamycin	50
Carbenicillin	100
Penicillin G	100
Tetracycline	12
Rifampicin	50
Vancomycin	10
Chloramphenicol	100
Colistin	5
Cefalexin	50

Supplementary Table S5. Statistics of clean genomic data obtained after fastq trimming

Sample name	Clean Reads	Clean Base	Read Length	Q20 (%)	GC (%)
BAUL-RG-20F-R05-02	4,203,811	1,261,143,300	PE150	93.85	63.69
CARO-RG-8B-R23-01	4,057,029	1,217,108,700	PE150	93.51	69.56
CARO-RG-8B-R24-01	4,052,146	1,215,643,800	PE150	94.17	64.73
CROZ-RG-20F-R02-07	4,205,378	1,261,613,400	PE150	93.36	67.13
CROZ-RG-20F-R04-06	4,126,825	1,238,047,500	PE150	93.94	58.73
CROZ-RG-20F-R04-15	4,131,243	1,239,372,900	PE150	94.31	58.59
MONT-RG-20F-10-7-02	4,028,310	1,208,493,000	PE150	94.52	59.94
MONT-RG-20F-R14-05	4,035,850	1,210,755,000	PE150	93.9	59.94
TREG-RG-20F-10-5-01	4,020,368	1,206,110,400	PE150	93.89	59.88
TREG-RG-20F-10-6-01	4,014,265	1,204,279,500	PE150	93.98	59.85
TREG-RG-20F-R18-01	4,189,150	1,256,745,000	PE150	93.4	65.03

Supplementary Table S6. Analysis of Deviance Table (Type II Wald chisquare tests) on the antibiotic resistance coefficient of the 24 strains selected for antibiotic resistance assays. Phase indicates the exponential or stationary phase at which strains have been tested. The phase and the strain *factor* were analyzed as fixed variables. *P* value adjustment was estimated with Tukey method for comparing a family of 48 estimates.

	Chisq	*Df*	*Pr(>Chisq)*
**Gentamicin**			
Strain	393.653	23	**<2.2e-16**
Phase	28.092	1	**1.157e-07**
Strain:Phase	110.249	23	**2.280e-13**
**Ampicillin**			
Strain	171.0278	23	**<2.2e-16**
Phase	5.5523	1	0.01846
Strain:Phase	80.3465	23	**2.79e-08**
**Streptomycin**			
Strain	181.1994	23	**<2.2e-16**
Phase	0.9611	1	0.3269
Strain:Phase	103.8320	23	**3.047e-12**
**Kanamycin**			
Strain	195.614	23	**<2.2e-16**
Phase	29.224	1	**6.446e-08**
Strain:Phase	109.919	23	**2.607e-13**
**Carbenicillin**			
Strain	185.5520	23	**<2.2e-16**
Phase	0.0289	1	0.8651
Strain:Phase	76.0049	23	**1.391e-07**
**Penicillin**			
Strain	173.1999	23	**<2.2e-16**
Phase	2.1399	1	0.14351

Strain:Phase	34.4238	23	0.05927
**Tetracycline**			
Strain	458.8361	23	**<2.2e-16**
Phase	1.6341	1	0.2011
Strain:Phase	77.5157	23	**7.979e-08**
**Rifampicin**			
Strain	520.8060	23	**<2.2e-16**
Phase	0.7714	1	0.3798
Strain:Phase	78.6529	23	**5.239e-08**
**Vancomycin**			
Strain	568.624	23	**<2.2e-16**
Phase	11.026	1	**0.0008983**
Strain:Phase	121.100	23	**2.638e-15**
**Chloramphenicol**			
Strain	519.4214	23	**<2.2e-16**
Phase	5.5191	1	0.01881
Strain:Phase	175.8313	23	**2,00E-16**
**Colistin**			
Strain	507.401	23	**<2.2e-16**
Phase	24.529	1	**7.321e-07**
Strain:Phase	96.928	23	**4.750e-11**
**Cefalexin**			
Strain	162.230	23	**<2.2e-16**
Phase	18.782	1	**1.466e-05**
Strain:Phase	103.098	23	**4.090e-12**

SUPPLEMENTARY FIGURES

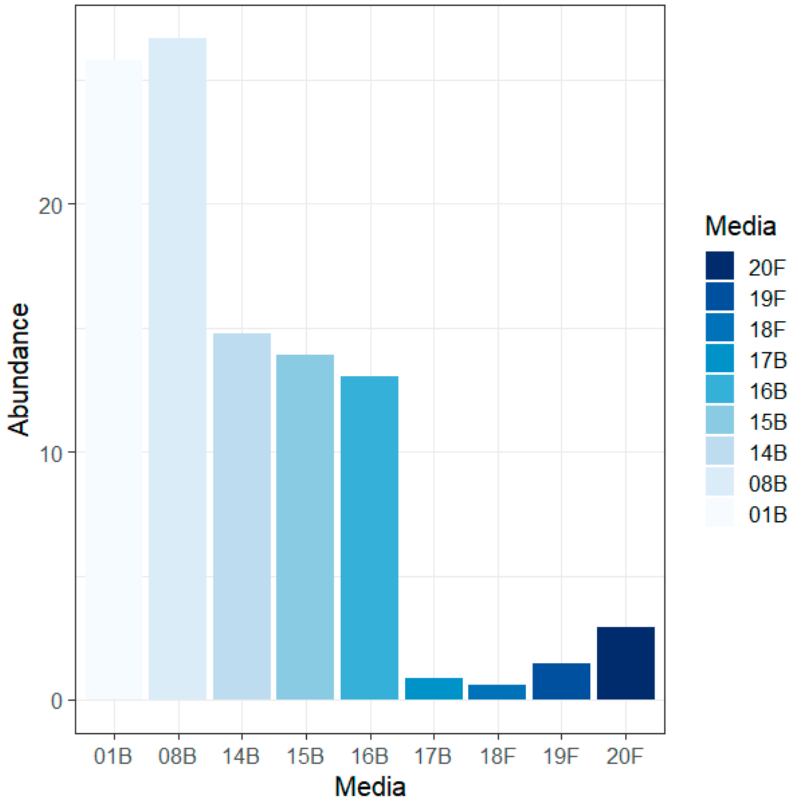

Figure S1. Number of species isolated for each medium. Bar plot is based on the abundance of each bacterial species (expressed in %) calculated by dividing the number of species recovered on each medium by the number of the total species recovered. Bleu scale color represents the different media used for bacterial isolation (see Supplementary Methods). Bar plot clearly shows that as observed for the abundance of the bacterial isolated for each medium, the 01B and 08B media recovered the maximum of the bacterial diversity.

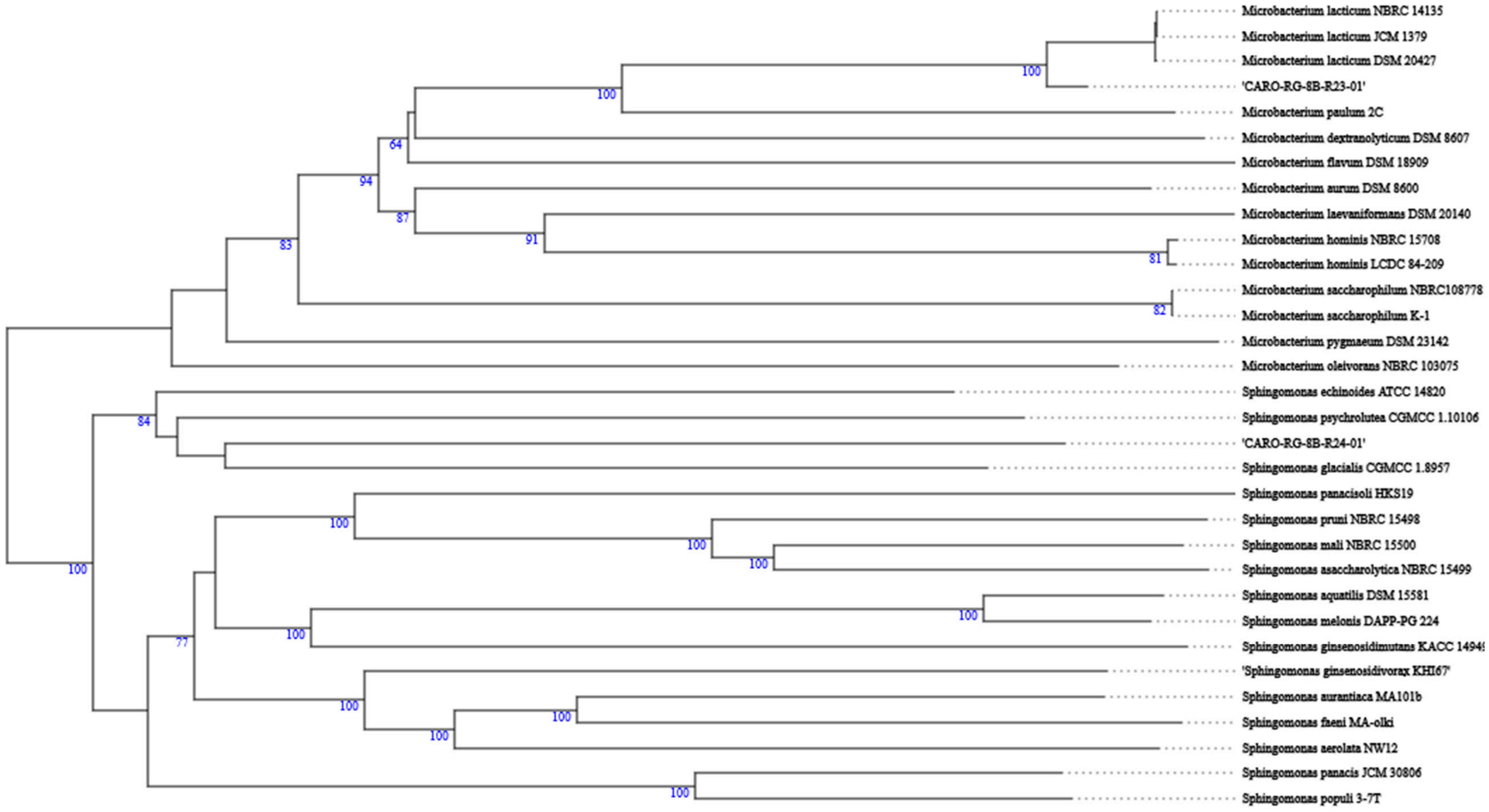

Figure S2. Phylogenetic tree based on the whole genome of the CARO-RG-8B-R24-01 and CARO-RG-8B-R23-01 strains obtained by the Type Strain Genome Service (TYGS).

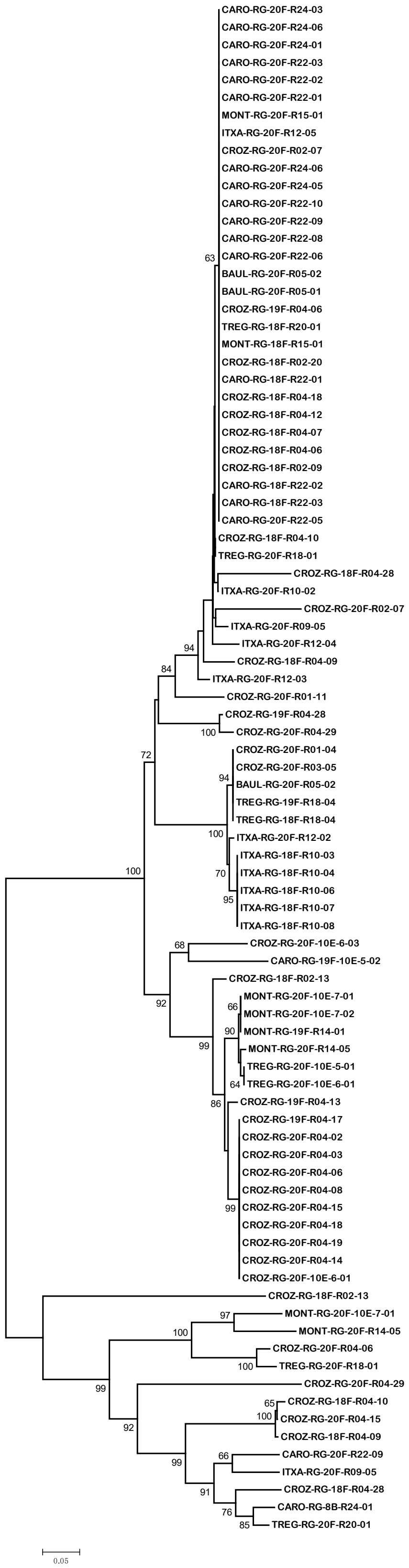

Figure S3. Phylogenetic tree based on the 16S sequences of strains isolated from media containing antibiotics.

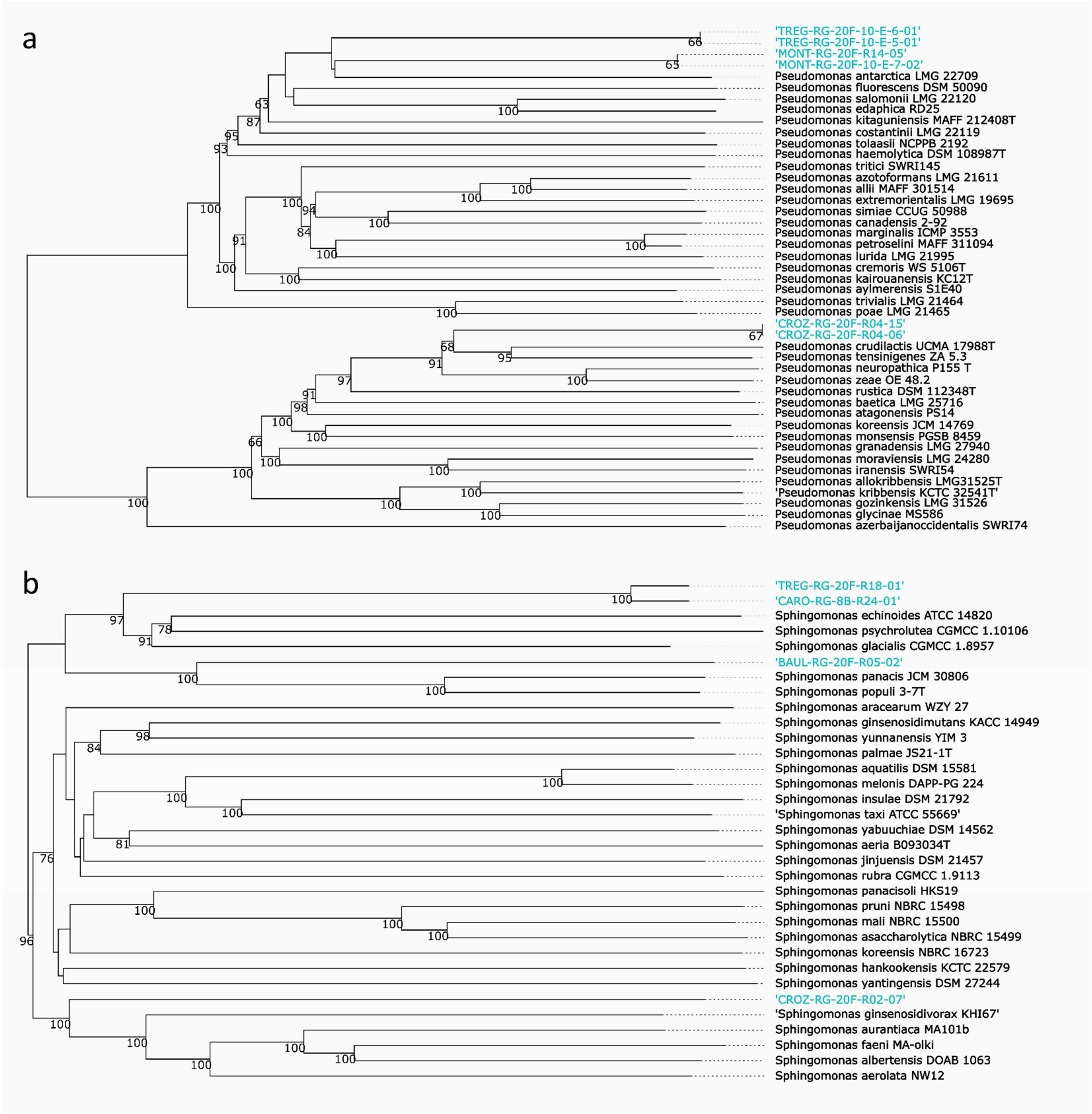

Figure S4. Phylogeny representing CROZ-RG-20F-R04-06, CROZ-RG-20F-R04-15, MONT-RG-20F-10-E-7-02, MONT-RG-20F-R14-05, TREG-RG-20F-10-E-5-01 and TREG-RG-20F-10-E-6-01 bacterial strains (a) and BAUL-RG-20F-R05-02, CARO-RG-8B-R24-01, CROZ-RG-20F-R02-07 and TREG-RG-20F-R18-01 (b) bacterial strains. Phylogenetic tree was inferred with FastMe 2.1.6.1 on the TYGS platform [56] from GBDP distances calculated from whole-genomes. The tree was rooted ad midpoint. Strains isolated from *R. geographicum* and the related tree branches are indicated in blue.

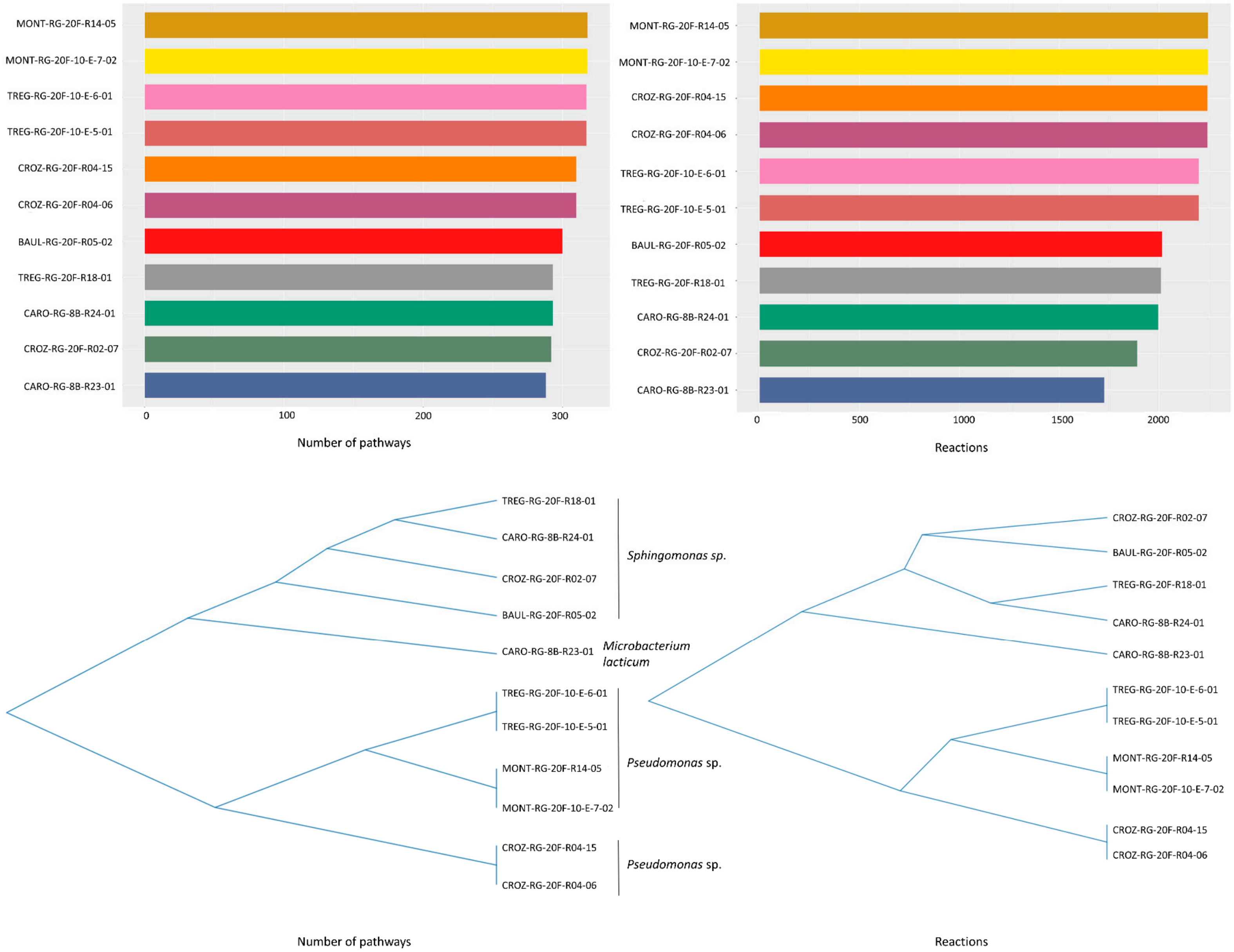

Figure S5. Network analysis based on genome functional annotation. Number of pathways (top left barplot) and number of reactions (top right barplot) based on the annotations of the 11 genomes. Clustering analyses based on the presence/absence of biological pathways (bottom left) and biological reactions (bottom right). The functional clustering is coherent with the taxonomical annotation of the strains. Plots were performed thanks to the MeCompR shiny application[2].

[2] https://lipm-gitlab.toulouse.inra.fr/lcottret/mecompr

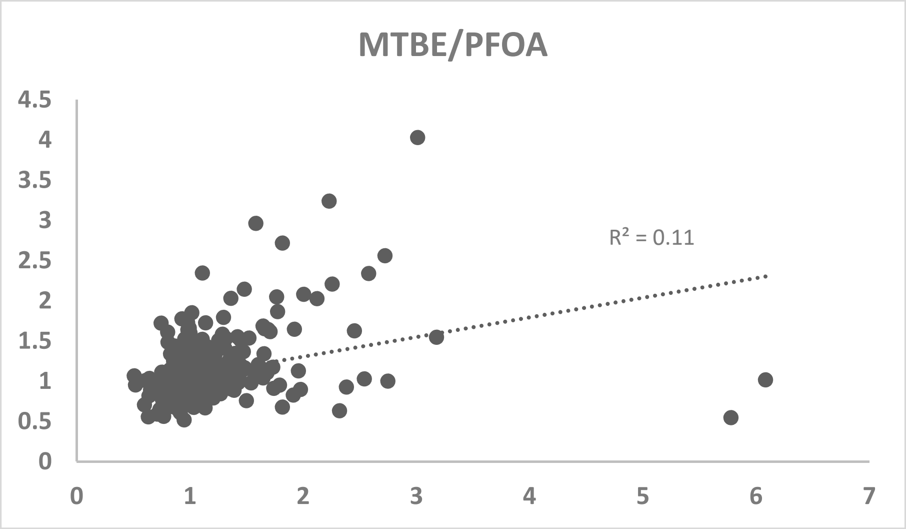

Figure S6. Correlation between Lsmeans values obtained from the POP tolerance experiment. *x*-axis represents Lsmeans values obtained from strains grew in presence of PFOA and *y*-axis represents Lsmeans values obtained from strains grew in presence of MTBE. The R^2 representing the correlation between the two variables is also reported.

References

1. Walters W, Hyde ER, Berg-Lyons D, Ackermann G, Humphrey G, Parada A, et al. Improved Bacterial 16S rRNA Gene (V4 and V4-5) and Fungal Internal Transcribed Spacer Marker Gene Primers for Microbial Community Surveys. *mSystems* 2015; **1**: e00009-15.

2. Li W, Godzik A. Cd-hit: a fast program for clustering and comparing large sets of protein or nucleotide sequences. *Bioinformatics* 2006; **22**: 1658–1659.

3. Ginestet C. ggplot2: Elegant Graphics for Data Analysis. *J R Stat Soc Ser A-Stat Soc* 2011; **174**: 245–245.

4. Aramaki T, Blanc-Mathieu R, Endo H, Ohkubo K, Kanehisa M, Goto S, et al. KofamKOALA: KEGG Ortholog assignment based on profile HMM and adaptive score threshold. *Bioinformatics* 2020; **36**: 2251–2252.

5. Meier-Kolthoff JP, Göker M. TYGS is an automated high-throughput platform for state-of-the-art genome-based taxonomy. *Nat Commun* 2019; **10**: 2182.

6. Machado D, Andrejev S, Tramontano M, Patil KR. Fast automated reconstruction of genome-scale metabolic models for microbial species and communities. Nucleic Acids Res 2018; **6**: 7542–7553.

7. King ZA, Dräger A, Miller P, Federowicz S, Lerman JA, Ebrahim A, Palsoon BA, Lewis NE. BiGG Models: A platform for integrating, standardizing and sharing genome-scale models. Nucleic Acids Res 2015; **44**: 515–522.
